# Digital Alloy-Grown InAs/GaAs Short-Period Superlattices with Tunable Band Gaps for Short-Wavelength Infrared Photodetection

**DOI:** 10.1021/acsphotonics.3c01268

**Published:** 2024-03-19

**Authors:** Bingtian Guo, Baolai Liang, Jiyuan Zheng, Sheikh Ahmed, Sanjay Krishna, Avik Ghosh, Joe Campbell

**Affiliations:** † Department of Electrical and Computer Engineering, University of Virginia, Charlottesville, Virginia 22904, United States; ‡ Department of Electrical and Computer Engineering, California NanoSystems Institute, University of California, Los Angeles, Los Angeles, California 90095, United States; § Beijing National Research Center for Information Science and Technology (BNRist), Tsinghua University, Beijing 100084, China; ∥ Department of Electrical and Computer Engineering, The Ohio State University, Columbus, Ohio 43210, United States

**Keywords:** absorption material, digital alloy, InGaAs, tunable band gap, optical constants, ellipsometry

## Abstract

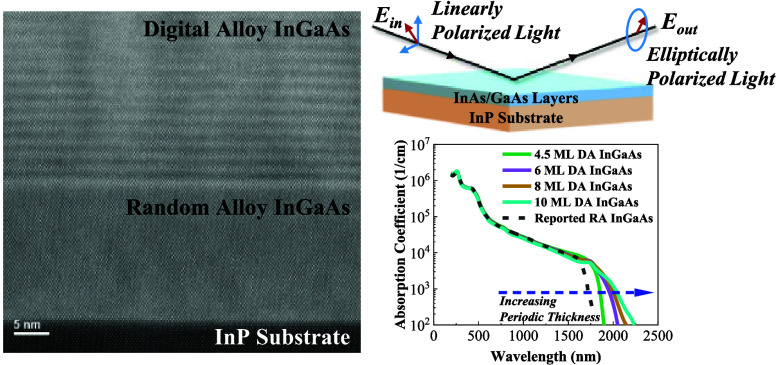

The InGaAs lattice-matched to InP has been widely deployed as the absorption material in short-wavelength infrared photodetection applications such as imaging and optical communications. Here, a series of digital alloy (DA)-grown InAs/GaAs short-period superlattices were investigated to extend the absorption spectral range. The scanning transmission electron microscopy, high-resolution X-ray diffraction, and atomic force microscopy measurements exhibit good material quality, while the photoluminescence (PL) spectra demonstrate a wide band gap tunability for the InGaAs obtained via the DA growth technique. The photoluminescence peak can be effectively shifted from 1690 nm (0.734 eV) for conventional random alloy (RA) InGaAs to 1950 nm (0.636 eV) for 8 monolayer (ML) DA InGaAs at room temperature. The complete set of optical constants of DA InGaAs has been extracted via the ellipsometry technique, showing the absorption coefficients of 398, 831, and 1230 cm^–1^ at 2 μm for 6, 8, and 10 ML DA InGaAs, respectively. As the period thickness increases for DA InGaAs, a red shift at the absorption edge can be observed. Furthermore, the simulated band structures of DA InGaAs via an environment-dependent tight binding model agree well with the measured photoluminescence peaks, which is advantageous for a physical understanding of band structure engineering via the DA growth technique. These investigations and results pave the way for the future utilization of the DA-grown InAs/GaAs short-period superlattices as a promising absorption material choice to extend the photodetector response beyond the cutoff wavelength of random alloy InGaAs.

## Introduction

Semiconductor quantum wells and superlattices have been widely used in various electronic and optoelectronic devices.^1−5^ With increasingly mature molecular beam epitaxy (MBE) growth techniques, atomic-level control of the epitaxial structure of multiple quantum wells (MQWs) has become achievable. The tuning of width, composition, and asymmetry of the MQW structure enables deterministic optical and electronic material parameters.^5−7^ The widely reported digital alloys (DAs) are essentially a short-period, multicomponent generalization of superlattices, where the superlattice period is reduced sufficiently that charge carrier wave functions integrate over many periods. They can improve the performance of optoelectronic devices, for example, circumventing the limitations imposed by miscibility gaps,^2,8^ engineering novel band structures,^9−12^ and providing a new method to achieve band gap tunability.^13,14^ For example, the limitations imposed by the miscibility gap encountered in the random alloy (RA) growth of Al*_x_*In_1–*x*_As_*y*_Sb_1–*y*_ lattice-matched to GaSb have been solved by using the DA growth technique.^2,7^ In addition to the investigation of Sb-based quaternary alloys, the DA growth technique can significantly modify the material characteristics of In_0.52_Al_0.48_As (hereafter InAlAs) and In_0.53_Ga_0.47_As (hereafter InGaAs) ternary materials lattice-matched to InP. The reduction of the excess noise in DA InAlAs avalanche photodiodes (APDs) has been experimentally and theoretically demonstrated.^13,15^ Compared to conventional RA InAlAs, wide band gap tunability is also obtained for DA InAlAs with different periodic structures.^14^ These new features of DA InAlAs have the potential to enhance the performance of APDs, particularly those used for telecommunications.

Like InAlAs, the ternary alloy InGaAs can be grown lattice-matched to InP. The RA InGaAs, which has a nominal band gap energy at 0.74 eV, has been widely used as an absorption material in photodetectors for a wide range of applications in the near-infrared and short-wavelength infrared regions (SWIR).^16−20^ Although Rockwell et al.^6^ have shown that the cutoff wavelength of 10-monolayer (ML) DA InGaAs APDs can be increased to be greater than 1900 nm, the reported results are primarily limited to the investigation on the single periodic structure at the APD level, and there is no systematic material investigation of DA InGaAs with different periodic structures. Furthermore, the previous growth of DA InGaAs p^+^-i-n^+^ APDs^6^ requires the addition of bismuth (Bi), which is not a necessary composition in the growth of other similar ternary materials such as DA InAlAs.^14^ Therefore, it is hard to conclude whether the band gap engineering of DA InGaAs results only from the digital alloy structure or from both bismuth addition and the digital alloy structure.

The systematic investigation of DA InGaAs without bismuth is necessary due to its photodetection capability at the extended short-wavelength infrared (SWIR) spectral range (1700–2500 nm). Recently, more work has been done in this spectral range, such as imaging,^21^ optical communications,^22^ and light detection and ranging (LiDAR),^10^ where conventional RA InGaAs cannot satisfy the application requirements and DA InGaAs could be a promising absorption material.

In addition to DA InGaAs, there are absorption material options such as InAs,^23^ Hg_0.7_Cd_0.3_Te,^24^ strained InGaAs, and traditional superlattices, but some inherent limitations exist for these materials. The operation of InAs and Hg_0.7_Cd_0.3_Te^23,24^ photodetectors requires cryogenic cooling due to the high dark current resulting from the narrow band gap, making it difficult to develop compact optical receivers based on these two materials. Strained InGaAs photodetectors are limited by the inherent high dark current originating from the lattice mismatch. In comparison to these three materials, DA InGaAs exhibits a tunable band gap and lattice matching in the extended SWIR spectral range. In comparison to type-II superlattice absorption materials such as InGaAs/GaAsSb superlattices,^25^ the DA InGaAs, short-period InAs/GaAs superlattices, has better interfaces, material uniformity, and growth quality, leading to lower dark current. DA InGaAs also has a type-I band alignment, making it possible to obtain a higher absorption coefficient. Therefore, it is expected that photodetectors with the DA InGaAs absorber have the potential to achieve a high performance in the extended SWIR spectral range.

In this work, we have studied a series of different DA InGaAs samples without using bismuth as a surfactant. They were characterized by scanning transmission electron microscopy (STEM), high-resolution X-ray diffraction (HR-XRD), atomic force microscopy (AFM), and photoluminescence (PL) measurements. The optical constants were then extracted via variable-angle spectroscopic ellipsometry (VASE) based on the Kramers–Kronig consistent basis spline (B-Spline) fitting approach. The band structures were simulated via an environment-dependent tight binding model^13^ to provide insights for the band gap tunability of the DA InGaAs. Increasing research interests in photodetection at the extended SWIR spectral range make such material investigation indispensable for its future utilization in research, commercial, and military applications.^16−19^

## Results and Discussion

### Sample Structures

**Figure 1:**
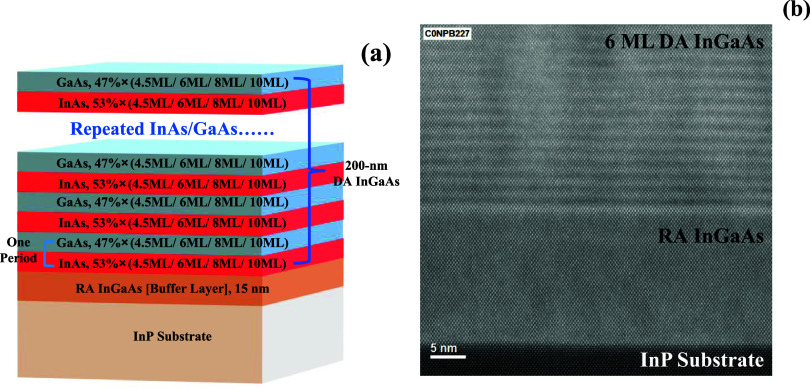
(a) Epitaxial structure of DA InGaAs samples with period thicknesses at 4.5, 6, 8, or 10 ML. (b) High-angle annular dark-field (HAADF) STEM image of the 6 ML DA InGaAs sample, where a 200 nm 6 ML DA InGaAs layer, 15 nm RA InGaAs layer, and InP substrate are sequentially shown from top to bottom.

The structure details of the investigated samples are shown in Figure 1a. Four DA InGaAs samples were grown with period thicknesses of 4.5, 6, 8, and 10 ML. For each sample, a 200 nm DA InGaAs layer was grown at 400 °C with repeated InAs/GaAs periodic structures. One control sample with a 200 nm RA InGaAs layer was also grown at 400 °C to determine the effect of the digital alloy structure on the band gap tunability.

Well-defined superlattice fringes in the top 200 nm DA layer were observed from the high-angle annular dark-field STEM image for the 6 ML DA InGaAs sample, as shown in Figure 1b. The DA InGaAs layer is uniform without dislocations, and the period thickness measured by STEM is approximately 6 ML.

### AFM Images

**Figure 2:**
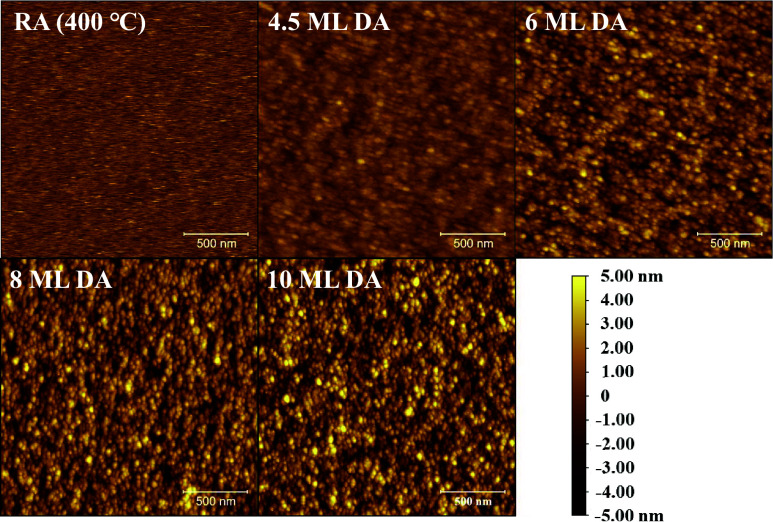
AFM images of RA InGaAs (400 °C), 4.5 ML DA InGaAs, 6 ML DA InGaAs, 8 ML DA InGaAs, and 10 ML DA InGaAs samples.

The morphology analysis of these samples was carried out using AFM at room temperature via a Bruker Dimension FastScan in tapping mode, as shown in Figure 2. The RA InGaAs (400 °C) sample exhibits a root-mean-square (RMS) roughness of 0.70 nm, and the DA InGaAs samples exhibit more rough films with spotty, strain relaxed-like surfaces, as demonstrated in the AFM images with RMS roughness at 0.93 1.27, 1.65, and 1.78 nm for 4.5, 6, 8, and 10 ML DA InGaAs samples, respectively. The RMS roughness gradually increases as the period thickness increases since the strain mismatch between InAs and GaAs increases. The rougher surface indicates that the material quality drops when the DA InGaAs sample is grown with a thicker periodic structure.

### HR-XRD

**Figure 3:**
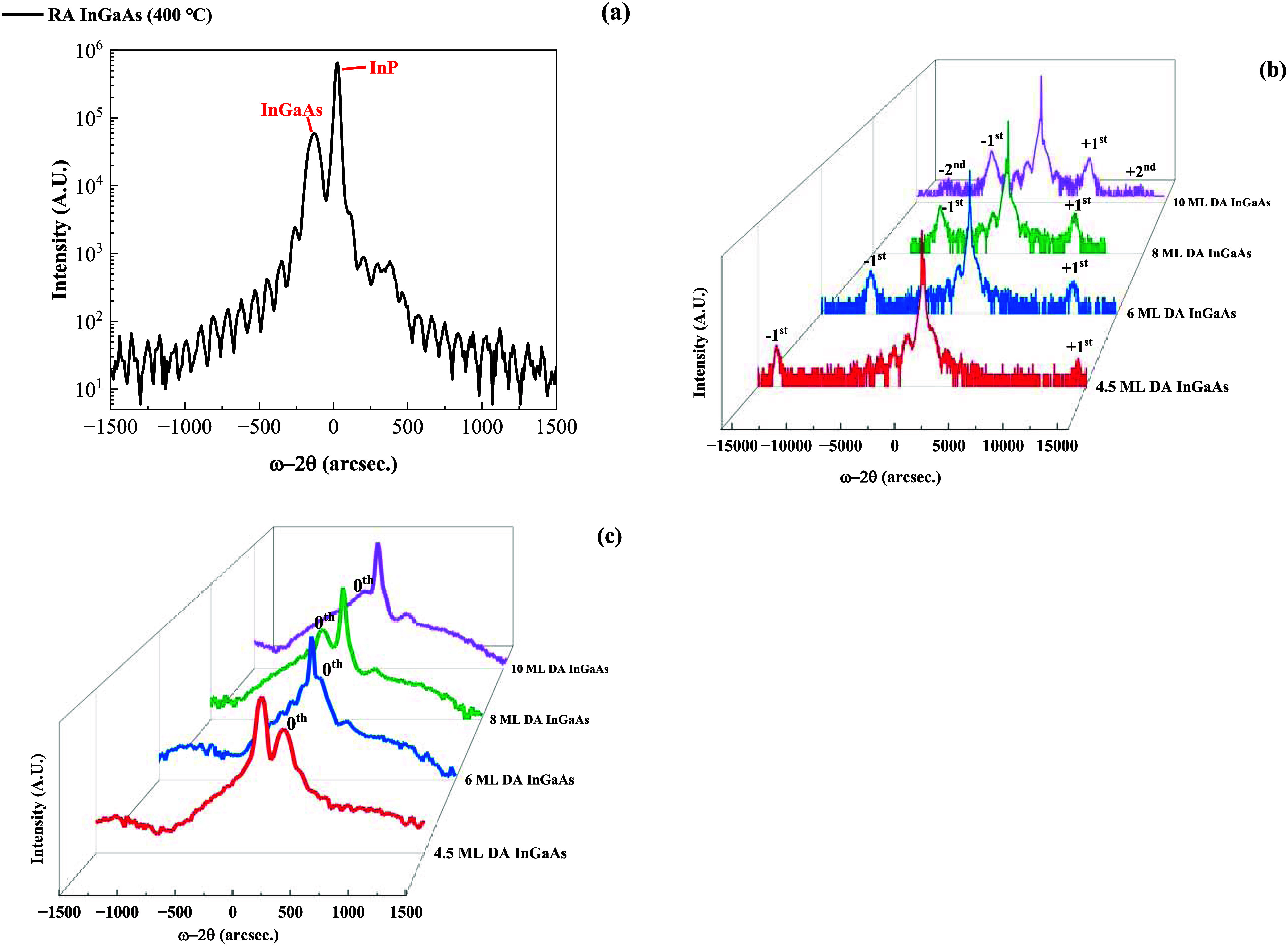
HR-XRD omega-2theta scans of (a) RA InGaAs (400 °C), (b) 4.5 ML DA InGaAs, 6 ML DA InGaAs, 8 ML DA InGaAs, and 10 ML DA InGaAs samples at room temperature. (c) Expanded HR-XRD spectra to show the splitting between the 0th peaks of the DA InGaAs samples and the InP substrate.

Figure 3a,b shows HR-XRD omega-2theta scans for all RA and DA samples. As for the RA InGaAs (400 °C) sample, clear thickness fringes can be resolved, and the composition is determined to be In_0.535_Ga_0.465_As, demonstrating a composition mismatch smaller than 0.5%. As for the DA InGaAs samples, the + first and – first satellite peaks can be observed for the 4.5, 6, and 8 ML samples, while the + second and – second satellite peaks can be observed for the 10 ML sample. These satellite peaks indicate the coherence and interface sharpness of the DA samples.

When the period thickness increases from 4.5 to 10 ML, the separation between + first and – first satellite peaks decreases and the intensities of the + first and – first satellite peaks increase, indicating enhanced coherence of the XRD diffraction signal for DA InGaAs with larger period thickness. Based on the satellite peak positions, the period thicknesses were estimated, matching well with the designed values.

Figure 3c expands the HR-XRD spectra to show the variation of the 0th peak of the DA InGaAs samples with respect to the InP substrate signal. The 0th peaks were measured to be at +200, +80, −180, and −220 arcsec relative to the InP substrate peak for 4.5, 6, 8, and 10 ML DA InGaAs samples, and the average indium compositions were then determined to be 51.9, 52.7, 54.1, and 54.4%, respectively. We conclude that the strain and average indium composition are impacted by the period thickness. To obtain thick DA InGaAs films with good material quality, it is necessary to carefully design the period thickness and optimize the growth conditions to balance the strain.

### PL Spectra

**Figure 4:**
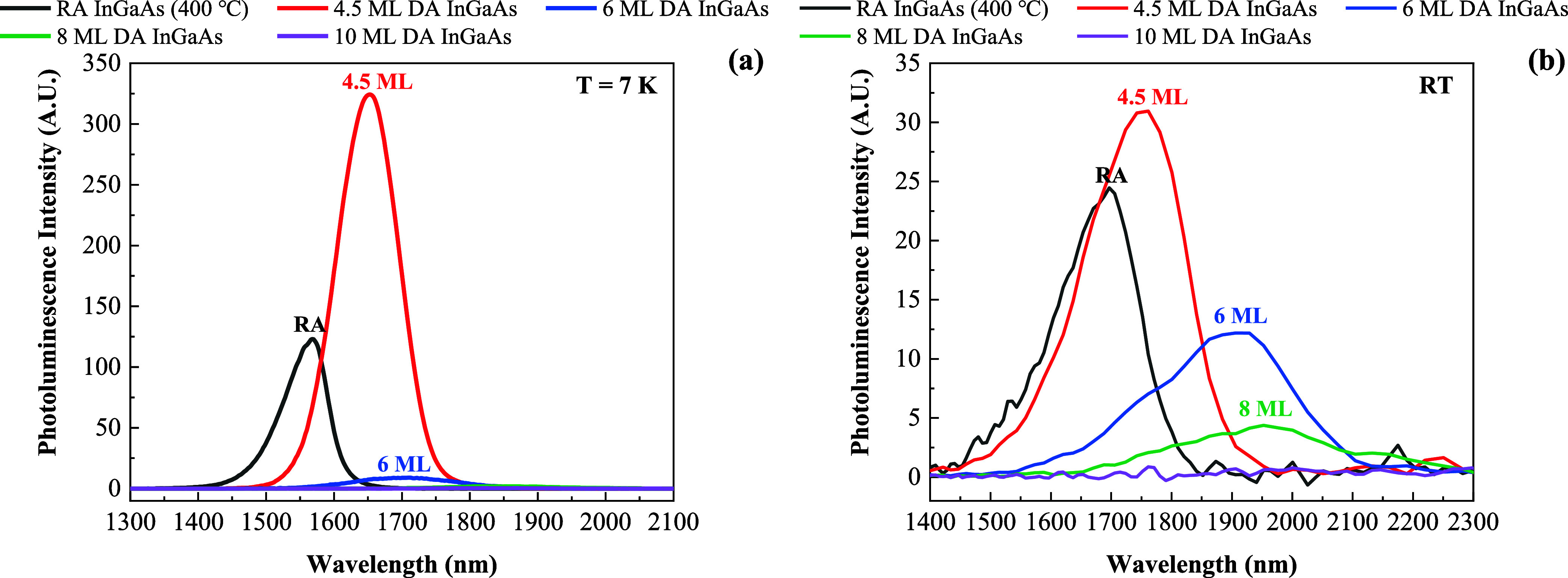
PL spectra of RA InGaAs (400 °C), 4.5 ML DA InGaAs, 6 ML DA InGaAs, 8 ML DA InGaAs, and 10 ML DA InGaAs samples at (a) 7 K and (b) room temperature. Normalized PL spectra are shown in Figure S1.

PL measurements were carried out on the RA InGaAs (400 °C) and DA InGaAs samples at cryogenic temperature and room temperature. The PL spectra measured at 7 K with an excitation intensity of 3000 W/cm^2^ and the PL spectra measured at 293 K with an excitation intensity of 1500 W/cm^2^ are shown in Figure 4a,b, respectively. Due to the high PL peak intensity of the 4.5 ML DA InGaAs, the PL spectra of the 8 and 10 ML DA InGaAs cannot be shown clearly. Therefore, the PL spectra at 7 K and room temperature were normalized, as shown in Figure S1. Some features can be obtained after careful analysis of these spectra.

Figure S1 indicates that the PL peaks exhibit a red shift as the period thickness increases. The PL peaks at 7 K are determined to be 1570 nm (0.790 eV), 1650 nm (0.751 eV), 1710 nm (0.725 eV), and 1820 nm (0.681 eV) and the PL peaks at room temperature are determined to be 1690 nm (0.734 eV), 1750 nm (0.708 eV), 1900 nm (0.653 eV), and 1950 nm (0.636 eV) for RA InGaAs (400 °C), 4.5 ML DA InGaAs, 6 ML DA InGaAs, and 8 ML DA InGaAs samples, respectively. The PL intensity of 10 ML DA InGaAs is too weak to determine the peak position accurately. The PL peak can be effectively shifted from 1690 nm (0.734 eV) for the RA InGaAs (400 °C) to 1950 nm (0.636 eV) for the 8 ML DA InGaAs at room temperature. Such a prominent red shift is explained by the band structure engineering originating from the DA growth, and the red-shift amplitude depends on the DA period thickness. Therefore, by only changing from RA growth to DA growth, the conventional InGaAs material system has been demonstrated with strong band gap energy tunability (∼260 nm) with the emission spectrum extended to ∼2 μm. It is lattice-matched to InP substrates, making it a promising absorber candidate for extended photodetection in the SWIR spectral range.

As shown in Figure 4, the 4.5 ML DA InGaAs sample has a higher PL intensity than that of the RA InGaAs sample and the other DA InGaAs samples exhibit dramatically decreased PL intensity as the period thickness increases. In particular, the PL emission from the 10 ML sample is barely discernible even at 7 K. It is likely that the DA structure of the 4.5 ML sample increases the quantum confinement of carriers, leading to enhanced carrier recombination and luminescence efficiency. However, when the period thickness increases from 6 to 10 ML, the samples have more defects and nonradiation trap centers, leading to a drop in the PL intensity.

**Figure 5:**
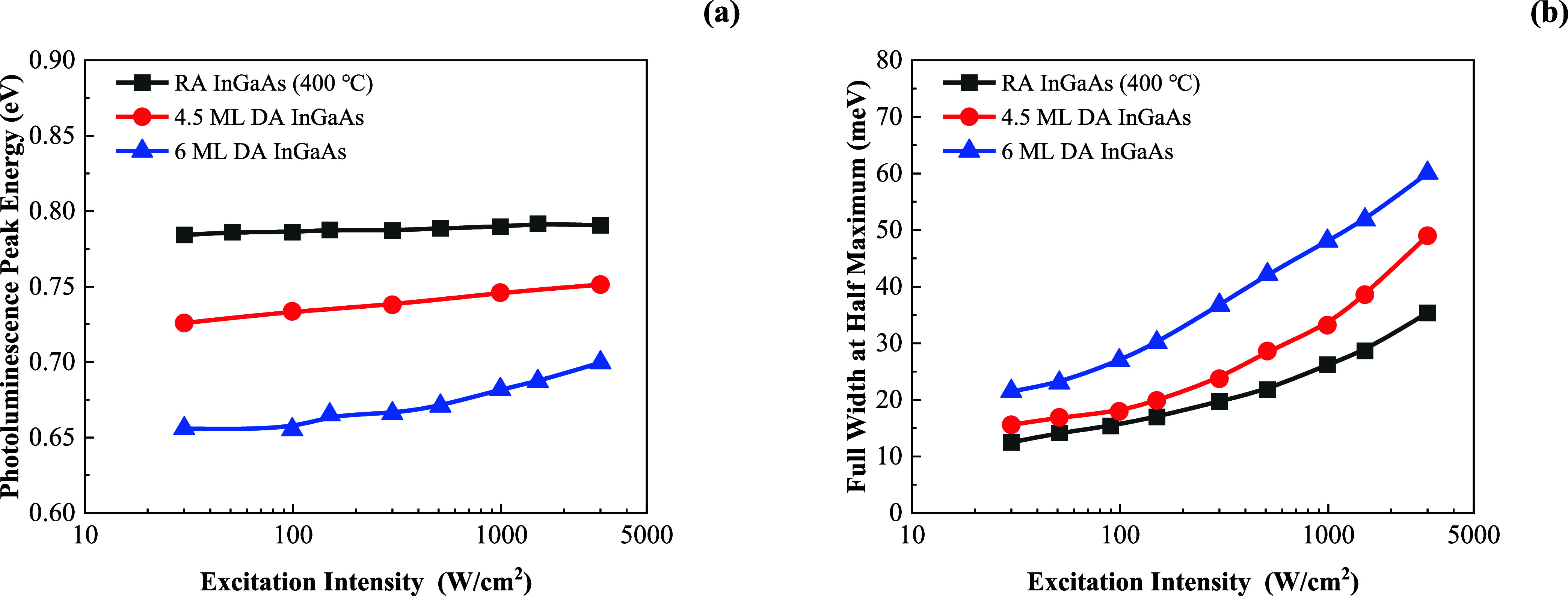
(a) PL peak energy and (b) fwhm measured under different laser excitation intensities at 7 K for RA InGaAs (400 °C), 4.5 ML DA InGaAs, and 6 ML DA InGaAs samples.

The excitation intensity-dependent PL spectra were measured, and the PL peak energy and full width at half maximum (fwhm) under different excitation intensities were extracted, as shown in Figure 5. For RA InGaAs (400 °C), 4.5 ML DA InGaAs, and 6 ML DA InGaAs samples, when the excitation intensity increases from 30 to 3000 W/cm^2^, the PL peaks shift 7, 26, and 44 MeV and the line width broadens 23, 33, and 39 MeV, respectively.

**Figure 6:**
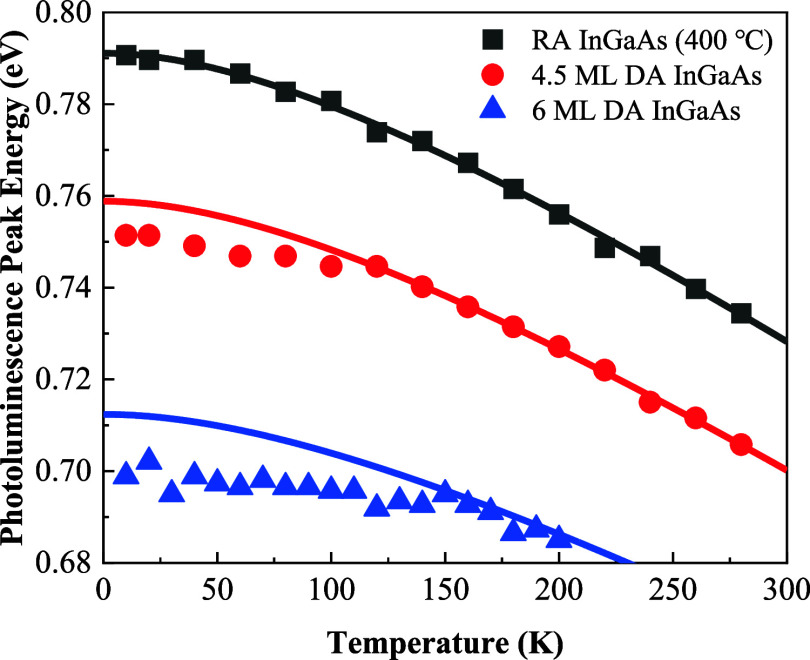
Temperature-dependent photoluminescence peaks (points) and Varshni fitting curves (solid lines) for RA InGaAs (400 °C), 4.5 ML DA InGaAs, and 6 ML DA InGaAs samples.

The temperature-dependent PL spectra were measured to further verify the carrier localization effect. Figure 6 shows the temperature-dependent PL peak energy from 10 to 280 K for RA InGaAs (400 °C), 4.5 ML DA InGaAs, and 6 ML DA InGaAs samples. The symbols represent the measured PL peak positions, and they can be fitted by the Varshni equation^26^
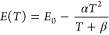
1where *E*(*T*) is the energy gap at temperature *T*, *E*_0_ is the energy gap at 0 K, and α and β are constants.

A good agreement was obtained for the RA InGaAs (400 °C) sample. For the 4.5 ML DA InGaAs sample, the measured and calculated band gap curves match well down to 120 K, and the discrepancy occurs in the temperature range from 10 to 120 K, where an “S” shape dependence of PL peak energy on temperature can be observed. Such an “S” shape behavior is generally regarded as a signature of strong carrier localization. A similar conclusion can be drawn for the 6 ML DA InGaAs sample, where the discrepancy occurs in the temperature range from 10 to 150 K, demonstrating a stronger carrier localization effect in comparison to the 4.5 ML DA InGaAs sample. In summary, the carrier localization effect becomes more pronounced as the period thickness increases.

### Extraction of Optical Constants via Ellipsometry

Optical parameters, including absorption coefficients and complex refractive indices, are significant for the design and optimization of optoelectronic devices, such as the calculation of the quantum efficiency,^27,28^ external or internal reflectivity,^29^ and photogenerated carrier injection profiles.^30−32^ The investigation of optical constants of optoelectronic materials facilitates their utilization in research and commercial applications.

Variable-angle spectroscopic ellipsometry was employed to study the absorption coefficients and complex refractive indices of DA InGaAs with different periodic structures. A Kramers–Kronig consistent B-Spline fitting method was chosen to extract the optical constants of the DA InGaAs due to its flexibility and accuracy of approximating optical constants.^27^ For the ellipsometry measurement, the polarization change (ρ) of the reflected light is expressed as the ratio of its parallel (*r*_*p*_) and perpendicular (*r*_*s*_) components
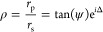
2where ψ and Δ are the amplitude ratio and the phase difference of parallel (*r*_p_) and perpendicular (*r*_s_) components of the reflected light, respectively.^33,34^

The RA InGaAs (400 °C) and DA InGaAs samples were measured with incident angles from 50 to 70° in 5° steps, as shown in Figure S2, and one extra sample with 215 nm RA InGaAs (485 °C) was also investigated. A good fitting between measured and simulated ψ and Δ was achieved at different angles for all of the samples. More fitting details can be found in the Supporting Information.

**Figure 7:**
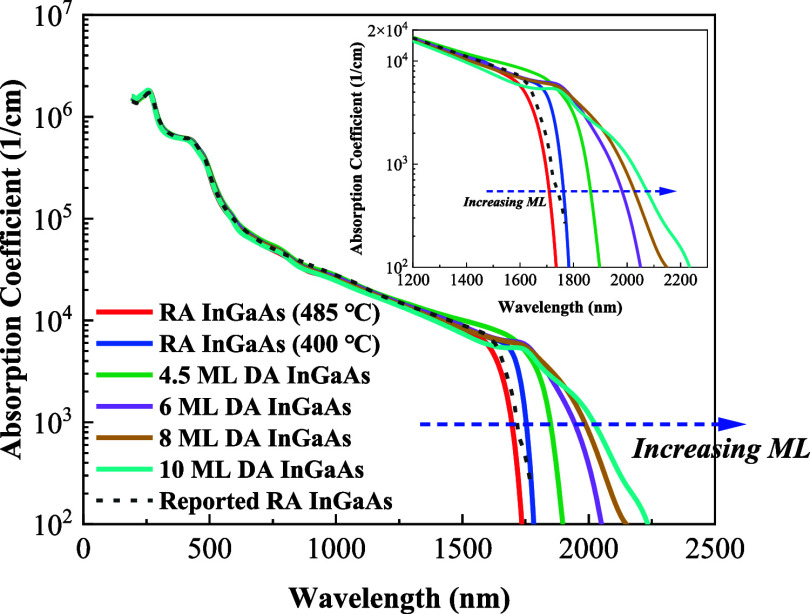
Comparison of absorption coefficients of RA InGaAs (485 °C), RA InGaAs (400 °C), 4.5 ML DA InGaAs, 6 ML DA InGaAs, 8 ML DA InGaAs, 10 ML DA InGaAs (solid lines), and literature-based RA InGaAs (dash line).^35^ The inset shows the absorption coefficient curves in the longer wavelength range.

The optical constants were successfully extracted from 193 nm to the region near the cutoff wavelength. The comparison of absorption coefficients between RA InGaAs (485 °C), RA InGaAs (400 °C), 4.5 ML DA InGaAs, 6 ML DA InGaAs, 8 ML DA InGaAs, and 10 ML DA InGaAs is shown in Figure 7 as solid lines. The absorption coefficients were determined to be 398, 831, and 1230 cm^–1^ at 2 μm for 6, 8, and 10 ML DA InGaAs. The dark dashed line represents the literature-based absorption coefficient of the typical RA InGaAs.^35^ There is a good agreement of absorption coefficients between the measured RA InGaAs (485 °C) and published RA InGaAs.^35^ The absorption coefficient curve in the region near the cutoff wavelength progressively shifts to a longer wavelength (lower energy) with increasing period thickness, the same trend as the PL results shown in Figure 4. The determination of the extinction coefficient (*κ*) and refractive index (*n*) is shown in Figures S3 and S4.

## Band Structure Simulation

To understand the underlying physics of the InGaAs band gap engineering, an accurate band structure spanning the full Brillouin zone is needed. We utilized the environment-dependent tight binding (EDTB) model,^36,37^ which can accurately compute the band structure of alloys. Material chemistry at interfaces and surfaces cannot be handled well by traditional tight binding models since they are only calibrated to the bulk band structure around the high-symmetry points.^37^ Thus, these models are not transferable to highly strained interfaces/surfaces that have a strong impact from the surrounding environment on the material properties. However, the EDTB model parameters are directly environment-dependent. These are calibrated to the state-of-the-art band structure generated using hybrid density functional theory^38^ along with their underlying orbital-resolved wave functions. The model incorporates environmental effects, such as strain- and interface-induced changes, by tracking neighboring atomic coordinates, bond angles, and bond lengths. Accurate band structures of unstrained and strained bulk III–V materials/alloys were used as fitting targets for our model. We have previously demonstrated that our model can match hybrid functional band structures of bulk, strained, and superlattice systems.^12,36,39^

**Figure 8:**
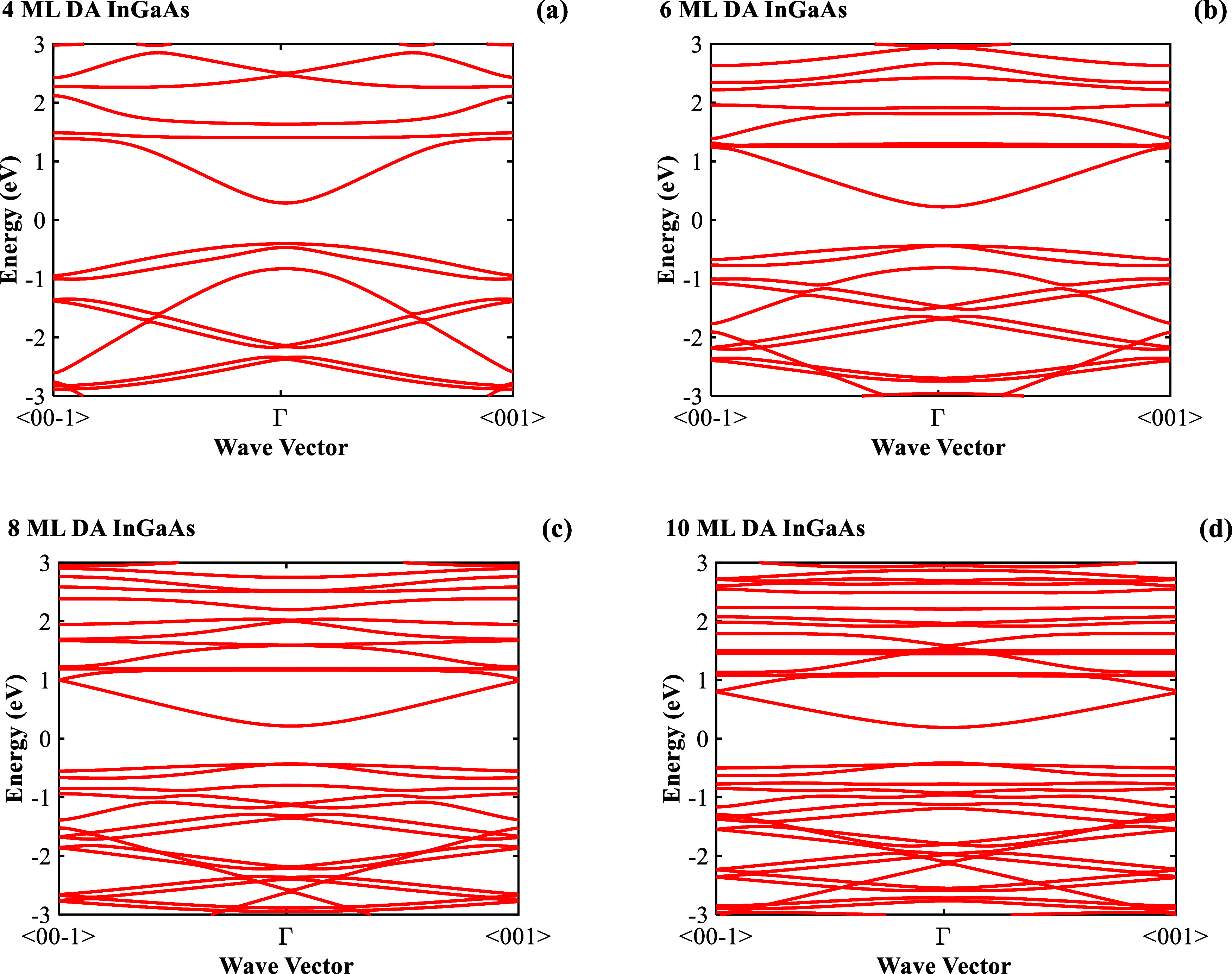
Band structures of (a) 4, (b) 6, (c) 8, and (d) 10 ML DA InGaAs.

**Figure 9:**
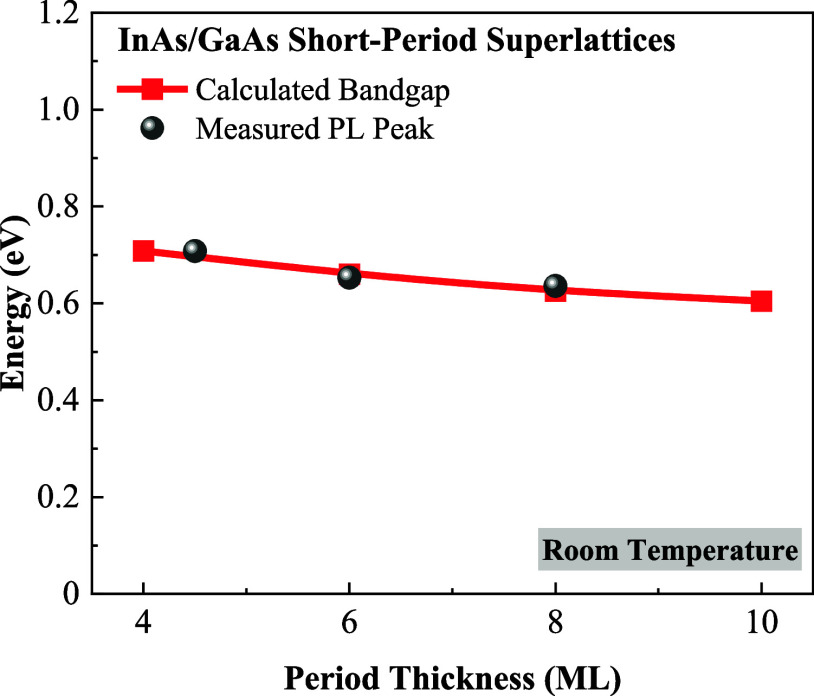
Comparison between the calculated band gaps and measured PL peaks at room temperature for DA InGaAs.

The band structures of 4, 6, 8, and 10 ML DA InGaAs were calculated based on the EDTB model,^13^ as shown in Figure 8. The calculated band gaps and measured PL peaks at room temperature agree well, as shown in Figure 9. The band structure of DA InGaAs is affected by the strain caused by the lattice mismatch between InAs and GaAs, and the strain experienced by InAs and GaAs varies with the period thickness. As the period thickness decreases, GaAs experiences stronger tensile strain and InAs experiences greater compressive strain.

The strain effects induced by the variation in period thickness play a vital role in determining the band gap of DA InGaAs. A thinner period thickness leads to a wider band gap overall, and this widening effect can be attributed to the increased tensile strain in GaAs. Our previous research indicates that the increased compressive strain in InAs has a relatively minimal impact on its band structure.^13^ Therefore, the band gap widens as period thickness decreases due to a stronger tensile strain experienced by GaAs. Conversely, increased thickness weakens the strain effect on both InAs and GaAs. As a result, the band gap of DA InGaAs decreases. This reduction occurs because the strain-induced band gap widening effect diminishes when the period thickness exceeds a critical threshold.

## Conclusions

A series of DA-grown InAs/GaAs samples with different period thicknesses ranging from 4.5 to 10 ML have been investigated. AFM, HR-XRD, and STEM measurements were carried out to reveal the morphologic and structural characteristics. The PL results exhibit a wide band gap tunability introduced by the DA structure. The optical constants of the DA InGaAs were extracted via ellipsometry based on the Kramers–Kronig consistent B-Spline fitting method, exhibiting the absorption coefficients of 398, 831, and 1230 cm^–1^ at 2 μm for 6, 8, and 10 ML DA InGaAs. For DA InGaAs with a thicker periodic structure, the corresponding absorption edge is shifted to longer wavelengths. The calculated band gap via an environment-dependent tight-binding model of DA InGaAs agrees well with the measured PL peak, and the simulated band structures are beneficial for a physical understanding of the effect of the digital alloy structure on band structure engineering. In summary, systematic investigations have been carried out for the DA InGaAs material system, and its absorption spectral range has the potential to be effectively extended to more than 2 μm, paving the way for future utilization of this conventional absorption material system grown by the digital alloy growth technique in SWIR applications.

## Methods

### Epitaxial Growth

The samples were grown on semi-insulating InP (001) substrates by a VEECO Gen-930 solid-source MBE system with valved cracking cells for dimeric As_2_ and Sb_2_. After removal of an oxide layer on the InP substrate surface, a 15 nm RA InGaAs buffer layer was grown at 485 °C, and then, a 200 nm DA InGaAs layer was grown at 400 °C. The growth of the DA InGaAs was undertaken as a short period superlattice (*d* ML) with rapidly alternating layers of GaAs (*d* ML × 47%) and InAs (*d* ML × 53%) in order to meet lattice-matching conditions on the InP substrate. In this research, four DA InGaAs samples were grown with period thicknesses of *d* = 4.5, 6, 8, and 10 ML. One sample with a 200 nm RA InGaAs layer was grown at 400 °C as a control sample. The growth temperature of the conventional RA InGaAs was generally chosen between 480 and 510 °C,^40^ but this temperature range would result in lateral composition modulation for the DA InGaAs growth.^6^ Therefore, a lower temperature of 400 °C was chosen for the 200 nm DA InGaAs layer, while the temperature was kept at 485 °C for the 15 nm RA InGaAs buffer layer growth. The lower growth temperature of 400 °C was used to reduce the lateral composition modulation in the DA InGaAs.

### Scanning Transmission Electron Microscopy

The TEM-ready samples were prepared using the in situ focused ion beam (FIB) lift-out technique on an FEI dual beam FIB/scanning electron microscope (SEM). The samples were capped with carbon and Pt prior to milling. The TEM lamellar thickness was ∼50 nm. The samples were imaged via an FEI Themis Z Advanced Probe aberration-corrected analytical TEM operated at 200 kV in STEM mode with a high-angle annular dark-field (HAADF) detector. The STEM probe size was about 1 Å nominal diameter.

### High-Resolution X-ray Diffraction

The HR-XRD pattern was acquired using a BEDE D1 system equipped with a Cu Ka radiation source with a wavelength of 0.15406 nm, operating at 40 kV and 40 mA. Double-crystal omega-2theta (ω–2θ) scans were conducted for all samples. The angle, denoted as ω, was systematically varied in the range of 29.5–34.5°, encompassing a broad spectrum to capture detailed insights into the InP peak and the superlattice zeroth, + first, and – first peaks of the DA layers.

### Photoluminescence Spectra

For the cryogenic-temperature PL spectra, the samples were mounted in an ARS-DE204 closed-cycle and variable-temperature (7–300 K) cryostat. The samples were excited by a 532 nm continuous-wave laser through a 50× objective lens. This objective lens also collected the PL signal and sent it onto the entrance slit of an Acton SpectraPro-2500i spectrometer connected to a Hamamatsu InGaAs photodetector for PL detection. For the room-temperature PL spectra, they were measured by a Nicolet 8700 FTIR using step-scan mode, while the samples were excited by a 671 nm continuous-wave laser through a 32× objective lens.

### Ellipsometry

Variable-angle spectroscopic ellipsometry was measured via an RC2 ellipsometer (J.A. Woollam Co.). A Kramers–Kronig consistent B-Spline fitting method was chosen to extract the optical constants, and the fitting was completed in CompleteEASE software.

## Data Availability

The data that support the findings of this study are available from the corresponding authors upon reasonable request.

## Supporting Information Available

Normalized PL spectra at 7 K and room temperature, description of the multilayer model building and the optical constant extraction of DA InGaAs via variable-angle spectroscopic ellipsometry, measured and simulated ψ and Δ of all samples, and extracted refractive indices and extinction coefficients of all samples (PDF)
