# Supplementary material for: Digital Alloy-Grown InAs/GaAs Short-Period Superlattices with Tunable Band Gaps for Short-Wavelength Infrared Photodetection
Source: ACS Photonics. 2024 Mar 19;11(4):1419–27. doi: 10.1021/acsphotonics.3c01268 (PMC11027153; doi:10.1021/acsphotonics.3c01268)
Supplement: Supplementary file 1 — ph3c01268_si_001.pdf [file ph3c01268_si_001.pdf]

Supporting Information

Digital Alloy-Grown InAs/GaAs Short-Period Superlattices with Tunable Bandgaps for Short-Wavelength Infrared Photodetection

Bingtian Guo,[1] Baolai Liang,[2,] Jiyuan Zheng,[3] Sheikh Ahmed,[1] Sanjay Krishna,[4] Avik Ghosh,[1] and Joe Campbell[1,*]*

[1]*Department of Electrical and Computer Engineering, University of Virginia, Charlottesville, Virginia, 22904, USA*

[2]*Department of Electrical and Computer Engineering, California NanoSystems Institute, University of California – Los Angeles, California, 90095, USA*

[3]*Beijing National Research Center for Information Science and Technology (BNRist), Tsinghua University, Beijing 100084, China*

[4]*Department of Electrical and Computer Engineering, The Ohio State University, Columbus, Ohio, 43210, USA*

[*]*Corresponding authors: bliang@cnsi.ucla.edu and jcc7s@virginia.edu*

1. Photoluminescence Spectra

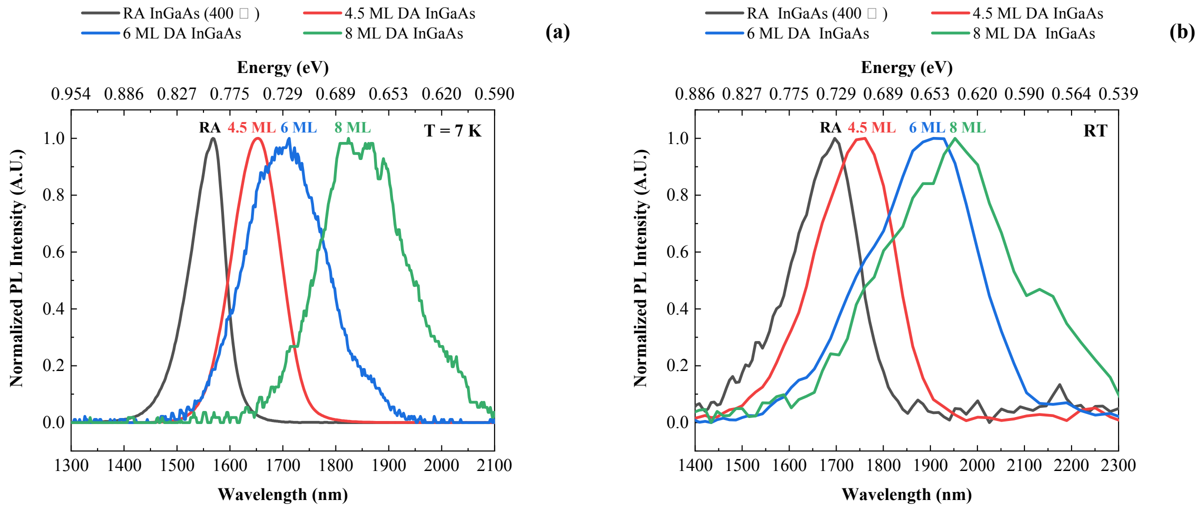

Figure S1. Normalized PL spectra of RA InGaAs (400 ℃), 4.5 ML DA InGaAs, 6 ML DA InGaAs, and 8 ML DA InGaAs at (a) 7 K and (b) room temperature.

2. Ellipsometry

The DA InGaAs and RA InGaAs (400 ℃) samples were measured with incident angles from 50° to 70° and 5° steps, and the measured ψ and Δ are shown in Figure S2. Then, a multilayer model is built to analyze

the polarization change of the reflected beam. As for the InP substrate, its optical constants come from the literature-based CompleteEASE library. As for the bottom 15-nm RA InGaAs layer grown at 485 ℃, since this layer has similar optical properties as 200-nm DA/RA InGaAs (400 ℃), it was essential to obtain precise optical constants of this 15-nm RA InGaAs layer in order to reduce the fitting uncertainty. Therefore, a new sample with a 215-nm RA InGaAs layer grown at 485 ℃ on the InP substrate was grown, and the optical constants of RA InGaAs (485 ℃) was firstly obtained from this sample via the Kramers–Kronig consistent B-Spline fitting method.

After the investigation of RA InGaAs (485 ℃), its optical constants were substituted into multilayer models of RA InGaAs (400 ℃), 4.5 ML, 6 ML, 8 ML, and 10 ML DA InGaAs samples to represent the bottom 15-nm buffer layer. The 200-nm investigated DA/RA InGaAs layers were modeled via the B-Spline method. In addition, a thin RA InGaAs oxide layer on the top was added into the consideration due to the samples' surface oxidization effect. Finally, the simulated ψ and Δ curves agree well with measured ψ and Δ curves, as shown in Figure S2. The optical constants were then successfully extracted. The absorption coefficients have been shown in Figure 5.

The absorption coefficient (α) and the extinction coefficient (κ) have the relationship of $\alpha = 4\pi\kappa/\lambda$, where λ is the wavelength, and the extinction coefficient and refractive index (n) have Kramers–Kronig relations. The comparison of the extracted refractive indices and extinction coefficients between 4.5 ML, 6 ML, 8 ML, and 10 ML DA InGaAs are shown in Figure S3, where a shifting trend can be observed as the period thickness increases for DA samples. Since the extinction coefficient and refractive index curves cover each other in Figure S3, these parameters of RA/DA InGaAs samples are shown separately in Figure S4 for a better view.

RA InGaAs (485 °C) **(a)**

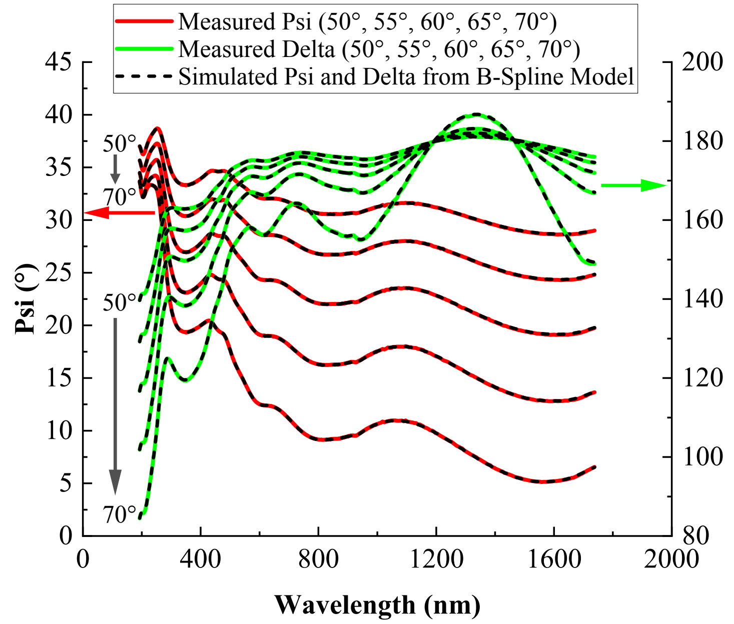

RA InGaAs (400 °C) **(b)**

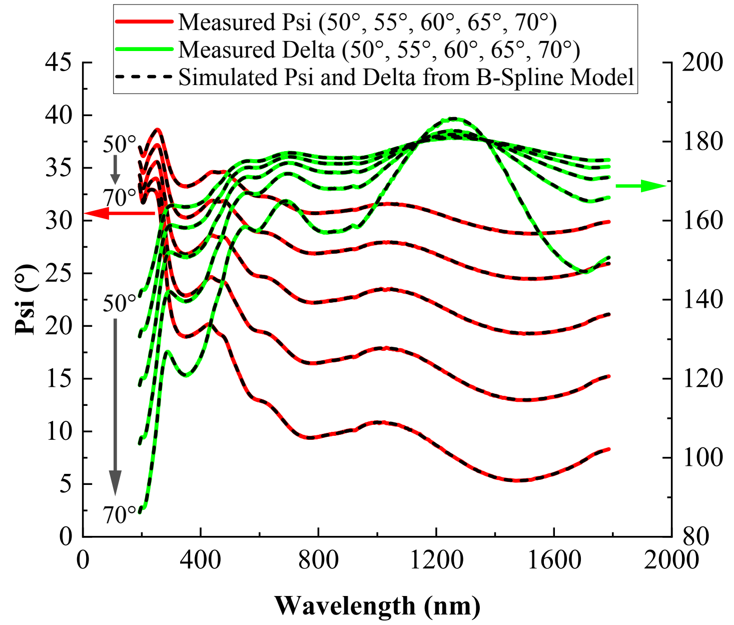

4.5 ML DA InGaAs

(c)

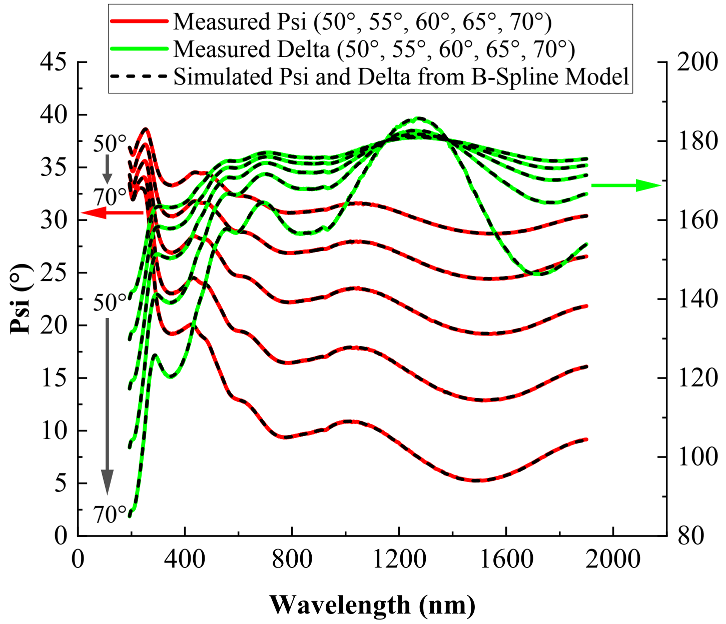

6 ML DA InGaAs

(d)

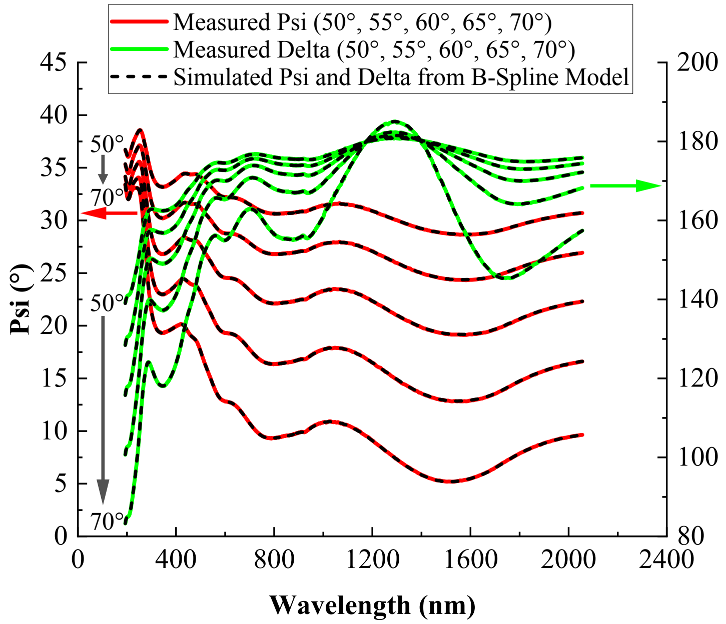

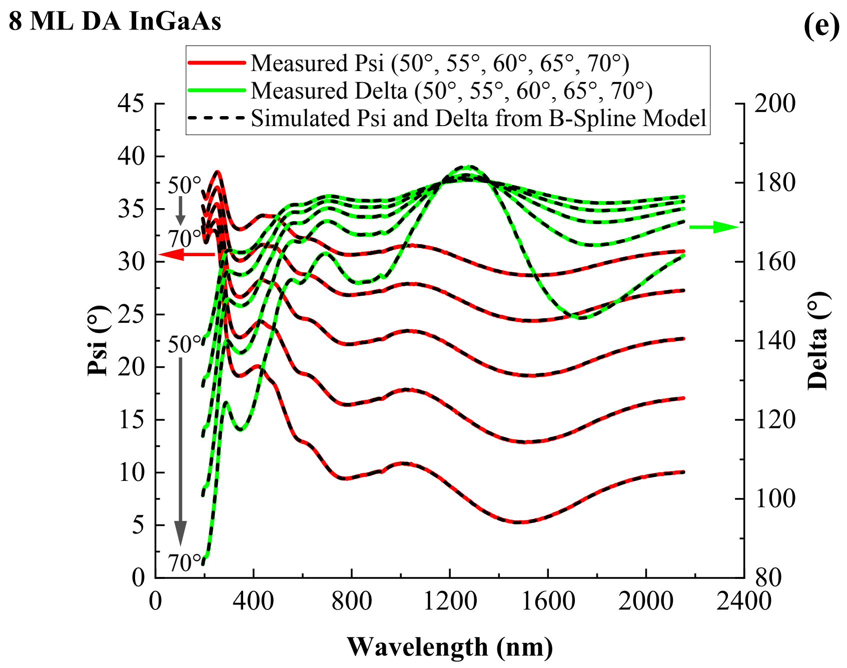

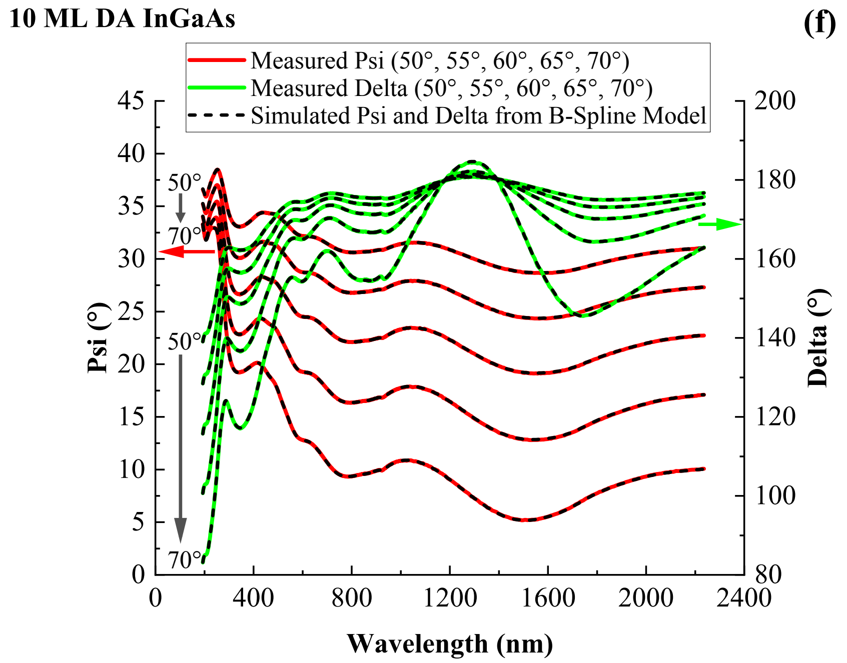

Figure S2. Variable-angle spectroscopic ellipsometry data with 50° - 70° incident angle of (a) RA InGaAs (485 ℃), (b) RA InGaAs (400 ℃), (c) 4.5 ML DA InGaAs, (d) 6 ML DA InGaAs, (e) 8 ML DA InGaAs, and (f) 10 ML DA InGaAs samples. Solid lines are measured ψ (red) and Δ (green), and black dash lines are simulated ψ and Δ based on the B-Spline fitting model.

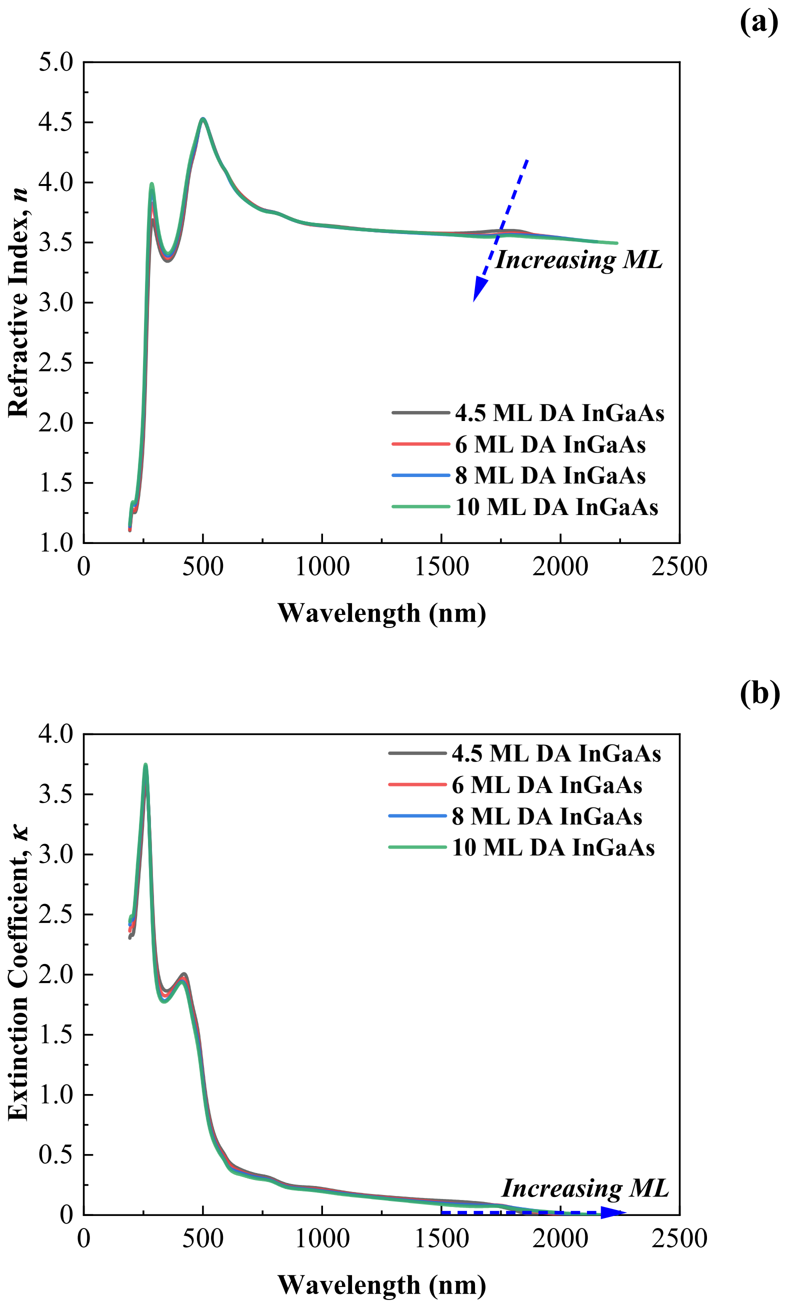

Figure S3. (a) Refractive indices and (b) extinction coefficients of 4.5 ML, 6 ML, 8 ML, and 10 ML DA InGaAs. Since the curves of refractive indices and extinction coefficients of different samples cover each other, these parameters of each sample are shown separately in Figure S4.

RA InGaAs (485 °C) **(a)**

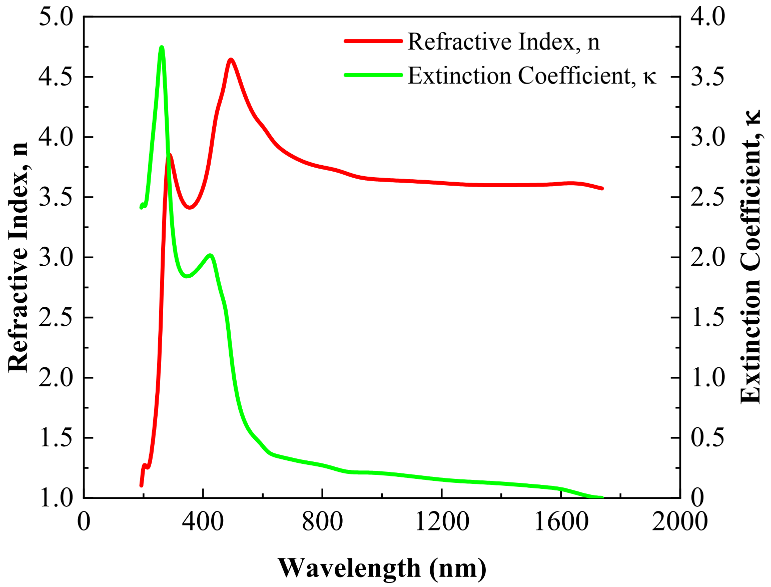

RA InGaAs (400 °C) **(b)**

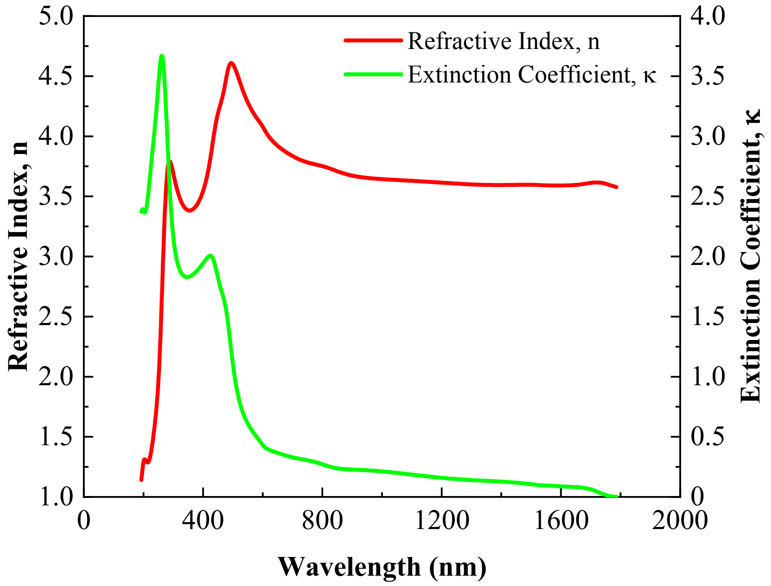

4.5 ML DA InGaAs **(c)**

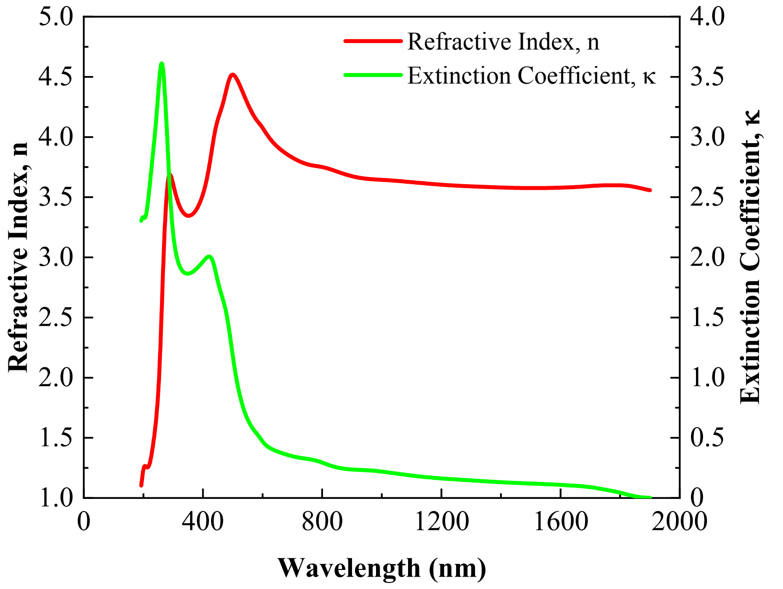

6 ML DA InGaAs **(d)**

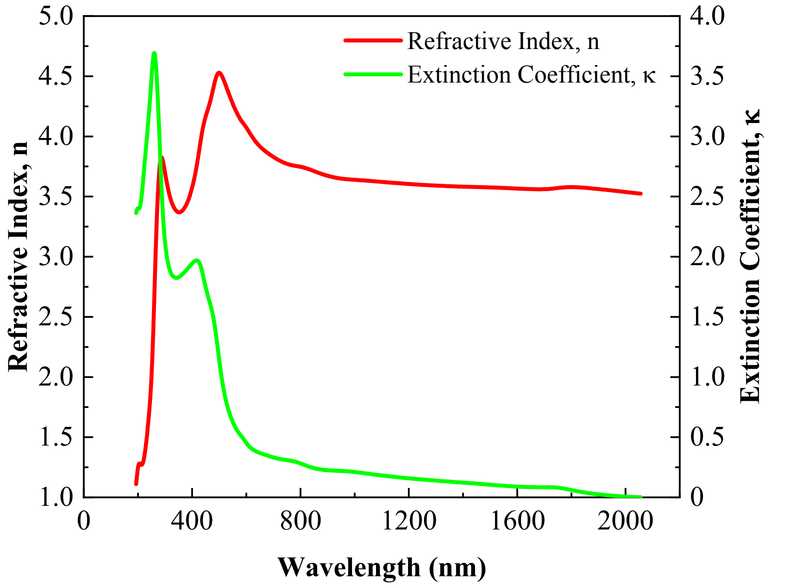

8 ML DA InGaAs (e)

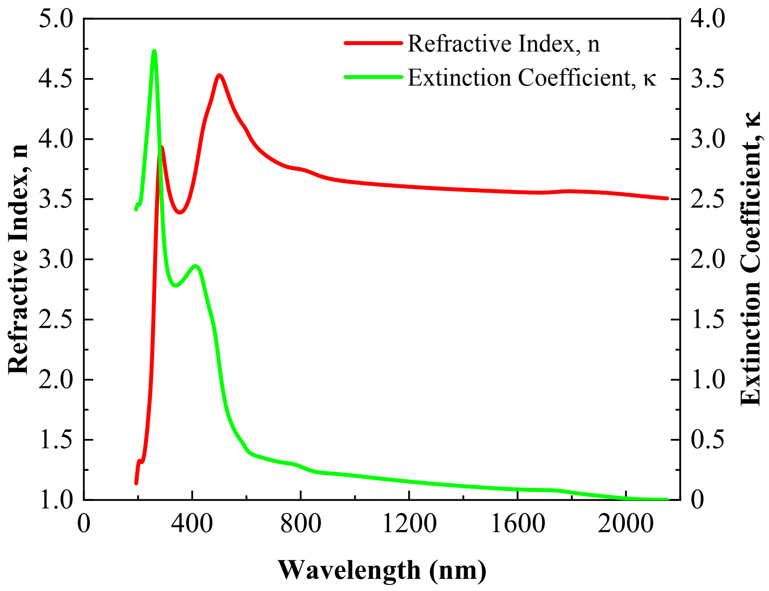

10 ML DA InGaAs (f)

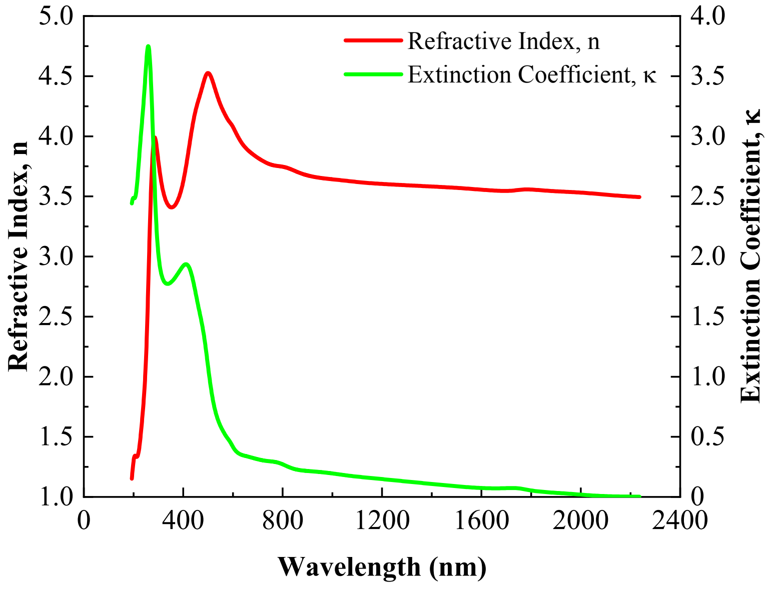

Figure S4. Refractive indices (red) and extinction coefficients (green) in a wide wavelength range of (a) RA InGaAs (485 °C), (b) RA InGaAs (400 °C), (c) 4.5 ML DA InGaAs, (d) 6 ML DA InGaAs, (e) 8 ML DA InGaAs, and (f) 10 ML DA InGaAs.
